# CXCL12 Promotes Stem Cell Recruitment and Uterine Repair after Injury in Asherman’s Syndrome

**DOI:** 10.1016/j.omtm.2017.01.001

**Published:** 2017-01-11

**Authors:** Gulcin Sahin Ersoy, Masoumeh Majidi Zolbin, Emine Cosar, Irene Moridi, Ramanaiah Mamillapalli, Hugh S. Taylor

**Affiliations:** 1 Department of Obstetrics, Gynecology and Reproductive Sciences, Yale School of Medicine, New Haven, CT 06520, USA

**Keywords:** stem cells, Asherman’s syndrome, uterus, fertility, CXCL12, AMD3100, CXCR4, intrauterine adhesions, cell therapy

## Abstract

Asherman’s syndrome is an acquired condition of uterine fibrosis and adhesions in response to injury that adversely affects fertility and pregnancy. We have previously demonstrated that bone marrow-derived mesenchymal stem cells (BMDSCs) contribute to uterine repair after injury and that stem cells supplementation improves fertility. Here, we demonstrate that CXCL12 is the chemokine that mediates stem cell engraftment and functional improvement using a murine model of Asherman’s syndrome. After uterine injury, we demonstrate that CXCL12 augmentation increased BMDSC engraftment and that the CXCL12 receptor (CXCR4) antagonist, ADM3100, blocked stem cell recruitment. CXCL12 reduced, whereas ADM3100 increased fibrosis. CXCL12 treatment led to improved fertility and litter size, whereas ADM3100 treatment reduced fertility and litter size. ADM3100 prevented optimal spontaneous uterine repair mediated by endogenous CXCL12 production, reducing pregnancies after injury in the absence of supplemental CXCL12 administration; however, ADM3100 treatment could be partially rescued by CXCL12 augmentation. CXCL12 or other CXCR4 receptor agonists may be useful in the treatment of infertility or adverse pregnancy outcomes in Asherman’s syndrome and other related uterine disorders.

## Introduction

Asherman’s syndrome (AS) is characterized by intrauterine adhesions or fibrosis following damage to the basal layer of endometrium.^1^ It arises most frequently following infection or trauma, especially in the postpartum period when estradiol levels are low.^2^, ^3^ Intrauterine adhesion and scarring often lead to amenorrhea, infertility, and various complications of pregnancy including placental abruption, preterm premature rupture of membranes, and malpresentation as a result of loss of normal endometrium.^4^, ^5^, ^6^ The prevalence of Asherman’s syndrome is reported as 13% in women undergoing routine infertility evaluation.^7^

Previously, several scaffolds such as biodegradable microporous synthetic materials, small intestine submucosa, and collagen loaded with collagen-binding human basic fibroblast growth factor^8^, ^9^, ^10^ were used as potential therapies in animal models of AS. However, all the available treatments are not consistently successful and have not been used in humans. Human therapies are confined to surgical remodeling of the uterine cavity. The final outcome of surgical therapy depends on the endometrial regeneration capability following restoration of uterine patency; however, women with Asherman’s syndrome display considerable inconsistency in endometrial growth.^11^, ^12^

Bone marrow-derived stem cell (BMDSC) therapy has recently been introduced as a potential treatment modality for Asherman’s syndrome. BMDSC transplantation may also prevent the occurrence of Asherman’s syndrome following uterine injury. We have previously shown that, post-injury, delivery of BMDSCs to mice dramatically improved reproductive performance in AS.^13^ BMDSCs express CXCR4, a chemokine receptor, which modulates cell migration. A vital role in stem cell mobilization and homing is mediated by the interaction of CXCR4 and its ligand, stromal-derived factor (SDF-1 or CXCL12).^14^ CXCL12 produced by the endometrium recruits BMDSCs to the uterus.^15^ We have previously demonstrated that CXCL12 is a chemoattractant for bone marrow (BM) stem cells and that the CXCR4 antagonist AMD3100 block the migration of BM-derived stem cells in vitro.^15^ In this study, we aimed to determine whether the migration of BMDSCs during endometrial repair in AS is enhanced by CXCL12, resulting in improved reproductive performance.

## Results

### Stem Cell Engraftment to the Uterus of Asherman’s Syndrome Is Mediated by CXCL12

**Figure 1:**
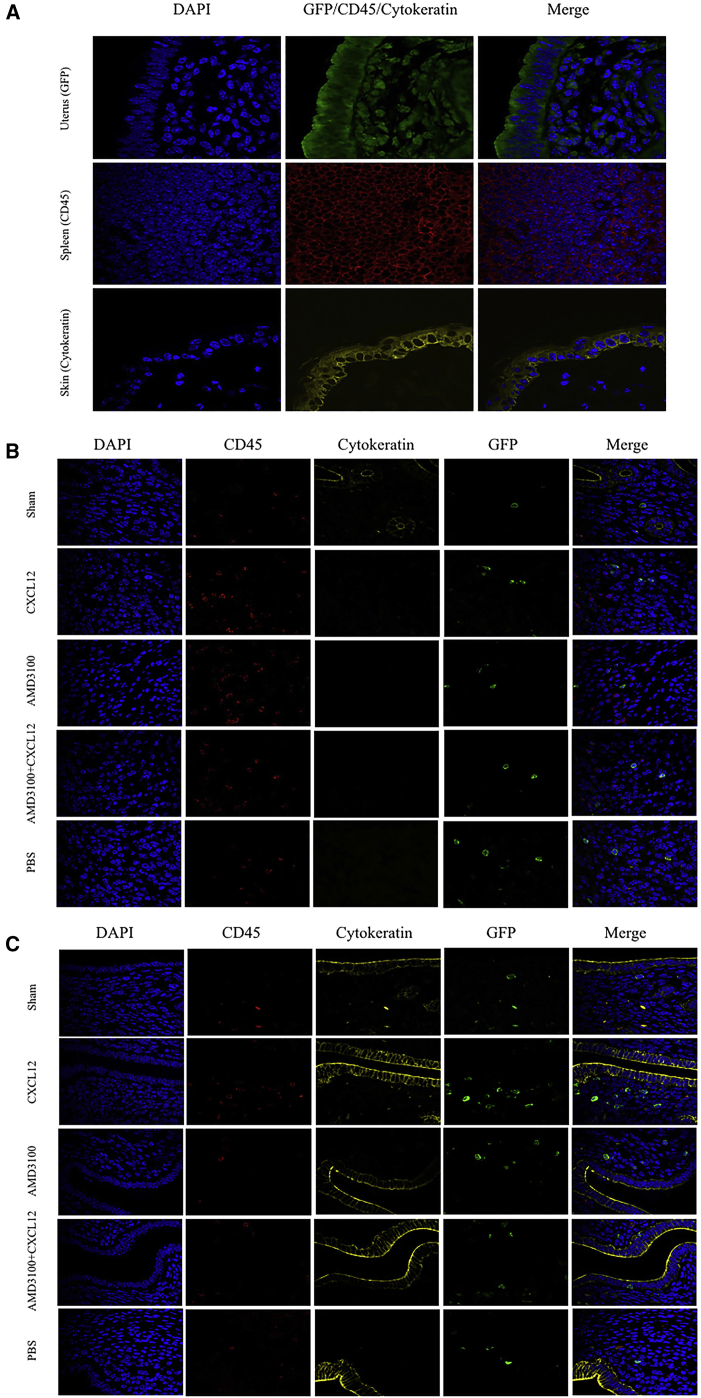
Immunofluorescence Staining of GFP, CD45, and Cytokeratin (A–C) Uterine tissue of GFP mice and spleen and skin tissue of wild-type mice were used as positive controls for GFP, CD45, and cytokeratin, respectively (A). Tissue sections from uteri of irradiated BM-transplanted mice are shown in (B) and non-irradiated, bone marrow-supplemented mice in (C). Tissues were stained with anti-GFP antibody (green) and co-stained with either anti-CD45 (pan-leukocyte marker) antibody (red) or cytokeratin (epithelial marker) antibody (yellow). Nuclei were stained by DAPI and are shown in blue. Fluorescence images were captured by confocal microscope. Here, we evaluate the recruitment of BM-derived stem cells after administration of GFP BM. Mice received either sham surgery (Sham) or induction of AS followed by administration of PBS control, CXCL12, AMD3100, or CXCL12 plus AMD3100. We evaluated the total number of BM stem cells (GFP^+^/CD45^−^) and the number of those cells that were contributing to the epithelial cell population (GFP^+^/CD45^−^/cytokeratin^+^) in the endometrium. The number of cells in each category is quantified in Figure 2.

We investigated the recruitment of BMDSCs to the uterus controls and AS in response to treatment with PBS, CXCL12, AMD3100, or CXCL12 plus AMD3100. Mice were evaluated after GFP^+^ BM transplant to assess BM cell incorporation after complete replacement of wild-type BM with GFP^+^ BM. Additionally, to eliminate the effect of radiation damage on the uterus that may affect uterine repair, we also tested the effect of these agents in animals that were not irradiated and supplemented with GFP^+^ BM (Figure 1B). As expected, without total BM replacement, recruitment of BM-derived cells to the uterus was much lower when BM was not completely replaced; however, both models clearly showed the effect of the CXCL12 and AMD3100 treatment.

Uteri from animals with AS and control mice were evaluated for GFP, CD45, and cytokeratin (CK) expression using immunofluorescence. The uterine tissue of GFP mice, and the spleen and skin tissue of wild-type mice were used as positive controls for GFP, CD45, and CK, respectively (Figure 1A). In Figure 1B, we assess the recruitment of stem cells (GFP^+^/CD45^−^) from BM to uterus after BM transplant and treatment with CXCL12 and/or AMD3100. As expected, when compared to sham surgery without uterine injury (sham), injured uteri recruited more stem cells to the uterus (sham versus PBS). Augmentation of CXCL12 led to increased recruitment of stem cells to the uterus. AMD3100 (CXCR4 antagonist) inhibited recruitment of stem cells to the uterus. Treatment with both CXCL12 and AMD3100 also prevented the enhanced recruitment demonstrated with CXCL12 alone.

In Figure 1C, we demonstrate the same relationship in the absence of BM transplant. The regimen used to ablate BM may affect uterine ability to recruit stem cells. Here, we treated the mice with intravenous (i.v.) injection of GFP^+^ BM without prior myeloablation. We see engraftment of BM-derived cells to the uterus at approximately 10% of the frequency seen when all bone marrow cells are GFP^+^. The same relative relationships are apparent as seen in the myeloablation and BM transplant model. More GFP^+^/CD45^−^ cells are recruited to the uterus after injury than in the sham controls. CXCL12 treatment leads to increased BM-derived stem cell recruitment, whereas AMD3100 blocks recruitment.

**Figure 2:**
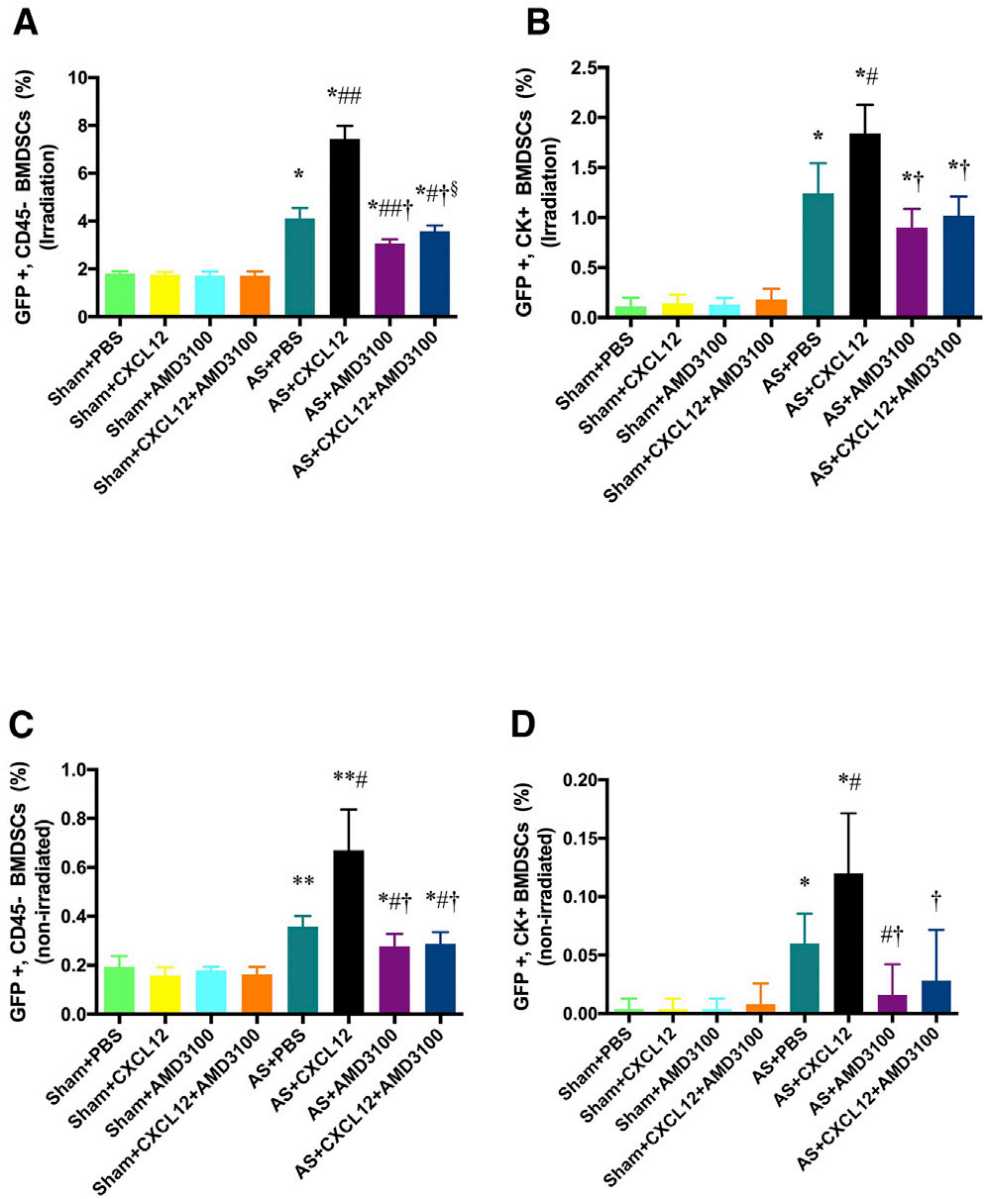
Recruitment of Stem Cells into the Uterus of Asherman’s Syndrome (A) Irradiation for myeloablation and GFP^+^ BM transplant. (B) Intravenous GFP^+^ BM supplementation. The sham and AS groups were treated with PBS, CXCL12, AMD31000, or CXCL12 plus AMD3100. The presence of GFP^+^/CD45^−^ cells was used to determine the number of engrafted BM-derived stem cell. In addition, GFP^+^/CD45^−^/CK^+^ cells were indicative of BM-derived epithelial cells. Uterine sections were analyzed by immunofluorescence staining as demonstrated in Figure 1 and quantified here. As expected, the mice receiving full BM transplant had far more BM-derived cells engrafting the uterus compared to animals receiving only transient BM administration. In either model, uterine injury (AS) recruits far more BM-derived stem cells and BM-derived epithelial cells compared to uninjured controls. CXCL12 treatment results in greater numbers of BM-derived cells recruited. AMD3100 (a CXCR4 antagonist) blocks CXCL12-mediated stem cell recruitment. AMD3100 blocks both endogenous CXCL12 function and the effects of supplemental CXCL12 administration. (A) The asterisk (*) denotes a statistically significant difference (p < 0.01) versus Sham plus PBS; ^#^p < 0.05, ^##^p < 0.01 versus AS plus PBS; ^†^p < 0.01 versus AS plus CXCL12; ^§^p < 0.05 versus AS plus AMD3100; and for (B), *p < 0.01 versus Sham plus PBS; ^#^p < 0.05 versus AS plus PBS; ^†^p < 0.01 versus AS plus CXCL12. Sham and AS treated groups supplemented with bone marrow. (C) CD45^–ve^ population of GFP^+ve^ BMDSCs. *p < 0.05, **p < 0.01 versus Sham plus PBS; ^#^p < 0.05 versus AS plus PBS; ^†^p < 0.01 versus AS plus CXCL12. (D) CK^+ve^ population of GFP^+ve^ BMDSCs. *p < 0.01 versus Sham plus PBS; ^#^p < 0.05 versus AS plus PBS; ^†^p < 0.05 versus AS plus CXCL12. Data are presented as mean ± SEM.

Figure 2 shows quantification and comparison of BM derived stem cells in the AS model. Figure 2A shows BM-derived stem cell recruitment after myeloablation and BM transplant with GFP^+^ BM. In the absence of injury to the uterus, approximately 2% of uterine cells were of BM stem cell origin (GFP^+^/CD45^−^). We excluded the leukocytes (CD45^+^) that are known to transiently populate the uterus and are expected to be of BM origin. In the absence of injury, CXCL12 and AMD3100 treatment had no effect. After uterine injury leading to AS, BM stem cell recruitment to the uterus was approximately doubled to 4%. Treatment with CXCL12 further increased recruitment of stem cells to approximately 7%. AMD3100 blocked stem cell recruitment below that of injury alone to a level that was not significantly different from that of an uninjured uterus. Furthermore, even augmentation of the CXCL12 signal was blocked by AMD3100. The CXCR4 antagonist was able to block stem cell recruitment driven by either endogenous or supplemental CXCL12. In Figure 2B, we assessed cytokeratin expression to identify the number of differentiated epithelial cells derived from BM stem cells (GFP^+^/CD45^−^/CK^+^). These cells represented a small fraction of the total cells in the uterus derived from BM stem cells. The majority of BM-derived cells were stromal cells. In the absence of injury, very few epithelial cells were generated from BM (<0.1%). After injury, greater than 1% of epithelial cells were engrafted from BM. CXCL12 significantly increased epithelial cell recruitment from BM. AMD3100 reduced the recruitment of epithelial cells in animals that were either supplemented with CXCL12 or that had only endogenous CXCL12 production. CXCL12 was required for BM stem cell recruitment and enhanced both stromal and epithelial cell engraftment to the injured uterus.

In Figures 2C and 2D, we show similar data using the AS model with BM augmentation without myeloablation. As expected, the percentage of engrafted cell is approximately 10% of that seen after myeloablation and complete BM replacement. However, the effect of treatments was proportionally the same as shown above in the myeloablation model. Injury resulted in a doubling of stem cell recruitment (Figure 2C). CXCL12 nearly doubled again the number of recruited stem cells. AMD3100 blocked the recruitment induced by either endogenous CXCL12 (PBS treatment) or CXCL12 supplementation. Similarly, bone marrow-derived epithelial cell engraftment was increased after injury and further increased by CXCL12 augmentation (Figure 2D). AMD3100 also blocked epithelial recruitment and engraftment induced by CXCL12.

### CXCL12 Reduced Fibrosis Formation in AS Mice

**Figure 3:**
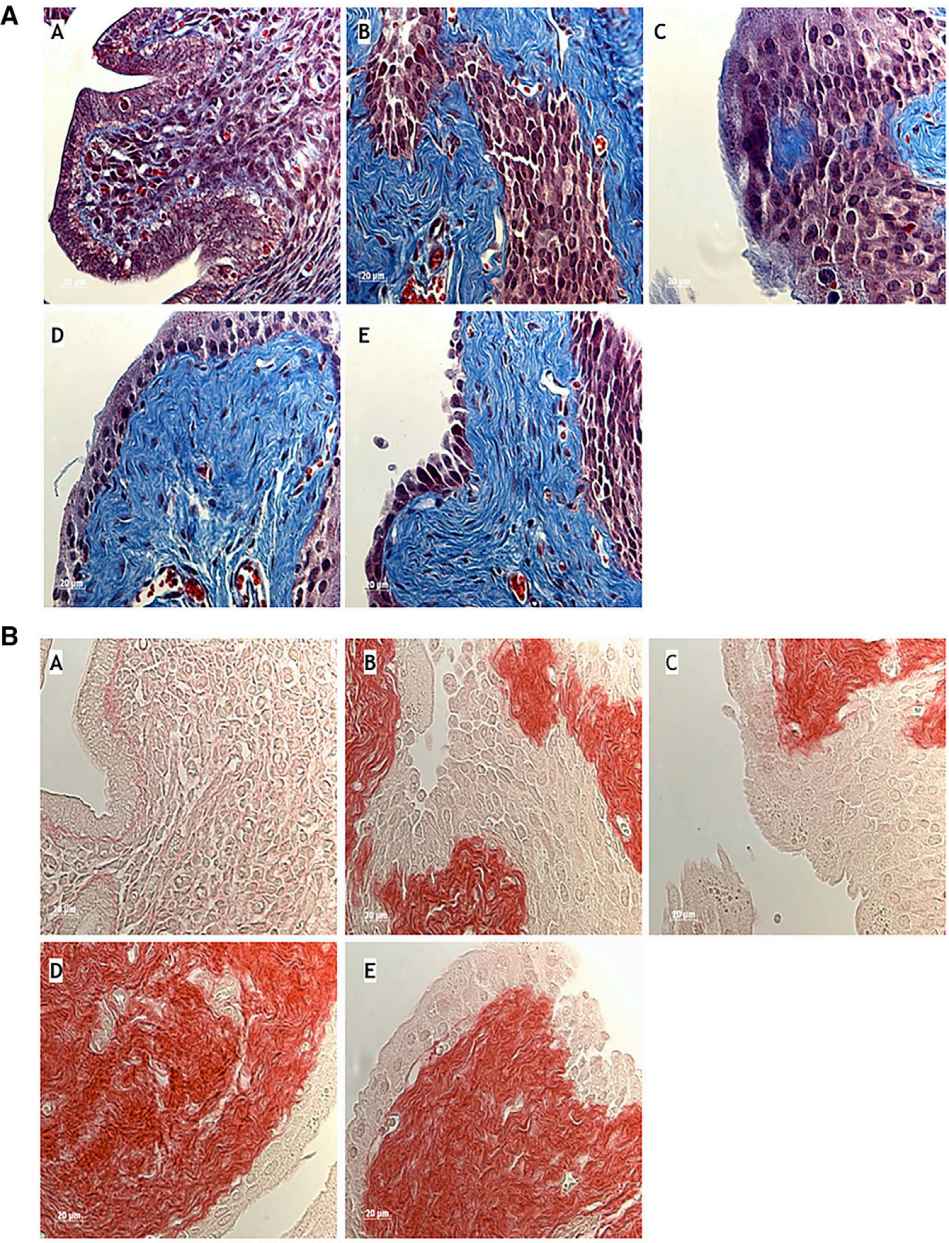
Fibrosis Histology after trichrome (A) and Sirius red (B) staining of uterine tissue in controls and the five groups with supplemental bone marrow transplantation at 3 weeks after Asherman’s syndrome modeling. Original magnification, 40×. (A) Uninjured controls; (B) AS with PBS control treatment; (C) AS with CXCL12 treatment; (D) AS with AMD3100 treatment; (E) AS with CXCL12 plus AMD3100 treatment. Fibrosis is increased after injury and reduced by CXCL12. AMD3100 treatment increases fibrosis, likely by blocking both endogenous CXCL12 (D) and CXCL12 treatment (E).

The extent of endometrial fibrosis due to collagen deposition in injury-induced mice treated with PBS or AMD3100 or CXCL12 plus AMD3100 as well as CXCL12 was determined by Masson’s trichrome and Sirius red staining of tissue sections from uterus of the above mice (Figures 3A and 3B, respectively). All comparisons were done with Sham-plus-PBS group because there was no significant difference between sham groups. Sirius red staining produced results similar to Masson’s trichrome staining. Significant fibrosis formation was found in AS mice compared to sham controls. Treatment with CXCL12 reduced fibrosis, whereas AMD3100 prevented the CXCL12-mediated reduction of fibrosis.

**Figure 4:**
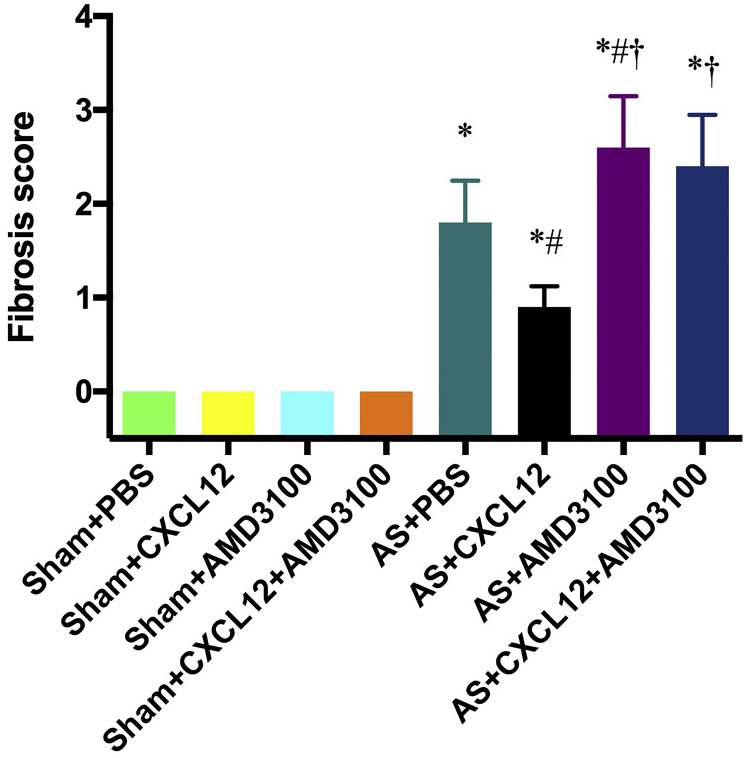
Fibrosis Is Reduced by CXCL12 Fibrosis scorings of uterine tissues in the eight groups with supplemental bone marrow (BM) transplantation at 3 weeks after Asherman’s syndrome modeling. AS increased fibrosis, whereas CXCL12 reduced fibrosis in AS. AMD3100 reversed the effects of endogenous or supplemental CXCL12 administration. *p < 0.01 versus Sham plus BM plus PBS; ^#^p < 0.05 versus AS plus BM plus PBS; ^†^p < 0.01 versus AS plus BM plus CXCL12.

The fibrosis was quantified and compared between treatments. Induction of AS resulted in significantly more fibrosis in all treatment groups. Fibrosis in AS mice treated with PBS, CXCL12, AMD3100, or CXCL12 plus AMD3100 were each significantly different from their respective sham controls (p = 0.004, p = 0.004, p = 0.005, and p = 0.005, respectively) (Figure 4). After CXCL12 treatment, fibrosis was decreased relative to PBS control (p = 0.015). AMD3100 treatment of AS, either alone or with additional CXCL12, led to increased fibrosis (p = 0.006 and p = 0.006, respectively) compared to both the PBS-treated control. The greatest difference in fibrosis in the AS mice was seen when the CXCL12-treated mice were compared to the AMD3100-treated mice. AMD3100 blocked both the endogenous CXCL12 as well as the supplemented CXCL12 administered to AS mice.

### CXCL12 Treatment Improved Pregnancy

**Figure 5:**
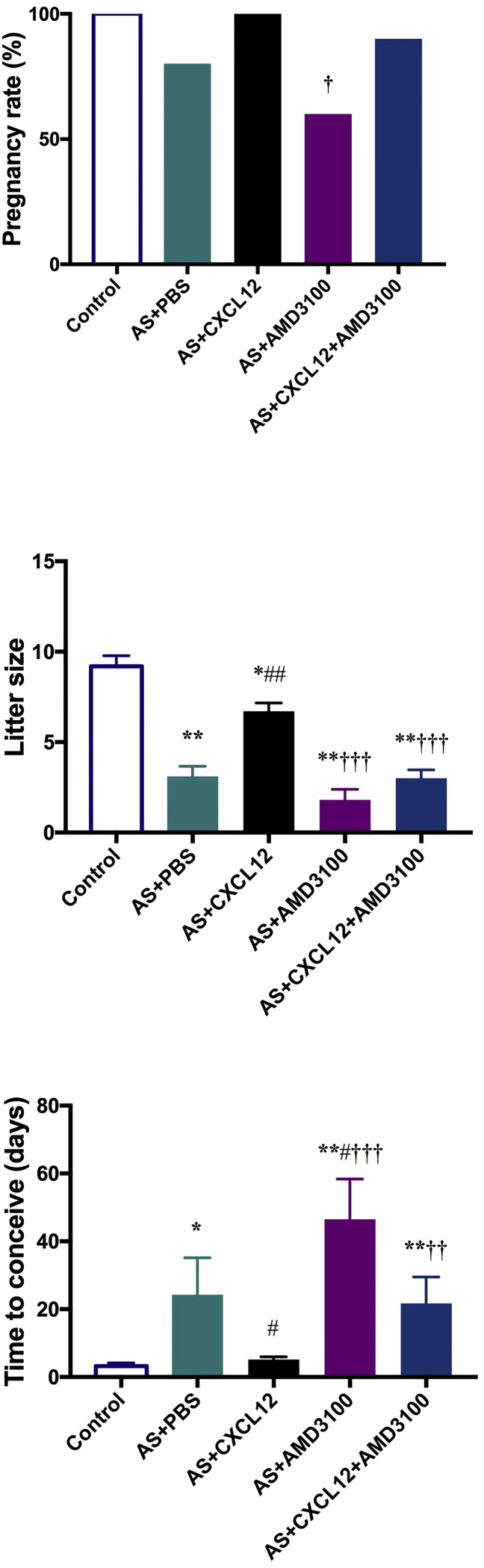
CXCL12 Restores Uterine Function Pregnancy outcomes among control and treated female groups were determined by mating with fertile wild-type males. Pregnancy rate is not significantly reduced in this mild AS model. However, blocking the ability of endogenous CXCL12 to spontaneously repair the uterus after injury by administration of AMD3100 did reduce pregnancy rate. Litter size and time to conceive are both significantly affected by injury in this model. CXCL12 treatment restored litter size and time to conceive. AMD3100 treatment blocked the effects of supplemental CXCL12. AMD3100 reduced pregnancy rate and litter size as well as increased time needed to conceive in this AS model. *p < 0.05, **p < 0.01 versus Control; ^#^p < 0.05, ^##^p < 0.001 versus AS plus PBS; ^†^p < 0.05, ^††^p < 0.01, and ^†††^p < 0.001 versus AS plus CXCL12. Data are presented as mean ± SEM.

The pregnancy rate, litter size, and time to conceive in AS mice with each of four different treatments, PBS, CXCL2, AMD3100, and CXCL12 plus AMD3100, were evaluated and compared to healthy control mice. We designed a model of mild AS to allow evaluation of therapies used for uterine repair that would be effective in the absence of BM transplant. As shown in Figure 5 (top panel), in this model where the cumulative pregnancy rate did not show any significant difference between controls and AS mice, presumably endogenous repair mechanisms prevented a significant decline in pregnancy rates. After treatment with CXCL12, pregnancy rate was restored to 100%; however, this was not significantly different than PBS treatment alone due to the small effect of mild AS on pregnancy rate in this model as just described. Strikingly, treatment with AMD3100 did lower pregnancy rate. AMD3100 blocks not only our CXCL12 treatment, it also blocks endogenous CXCL12 preventing uterine repair. Effective uterine repair after injury is mediated by CXCL12.

The litter size was significantly smaller (p = 0.002) in AS-induced mice compared to sham control mice as shown in Figure 5 (center panel). CXCL12 treatment significantly improved litter size (p < 0.001). In contrast, treatment with AMD3100 resulted in a decreased litter size compared to either the control mice or the CXCL12-treated AS mice (p = 0.002 and p < 0.001, respectively). These results show that CXCL12 treatment enhances litter size and that the CXCR4 antagonist AMD3100 blocked CXCL12-mediated uterine repair.

Additionally, as a measure of fertility, we evaluated the time to conceive in each treatment group. The control mice conceived rapidly, typically at the first mating. Induction of AS decreased fertility, leading to a prolongation of the time to conceive by nearly 5-fold. CXCL12 treatment significantly decreased the time to conceive in AS-induced mice and restored fertility to a level similar to controls (p = 0.02). AMD3100 treatment prevented repair and increased the time needed to conceive. AMD3100 blocked both endogenous CXCL12 and the ability of additional exogenous CXCL12 to fully repair the uterus. Here, the augmented CXCL12 was able to partially overcome the effects of AMD3100 but not to the point where fertility was restored to a level that was significantly better than no treatment.

## Discussion

Asherman’s syndrome is a common disease that affects fertility and pregnancy outcome. There are currently no effective treatments to prevent AS. Procedures that restore the patency of the uterine cavity are frequently unsuccessful in restoring normal fertility. Similarly, there are no effective therapies for women with a thin endometrium that results in infertility. We have previously shown that stem cells are recruited to repair the endometrium in response to injury.^16^ These cells are found in the circulation in limited quantities and often are insufficient to repair the uterus in the setting of overwhelming injury such as AS. Previously, we demonstrated that transfusion of large numbers of BM cells at the time of uterine injury was able to prevent infertility in a mouse model of AS.^13^ We have also shown that BM-derived stem cells are recruited to the uterus by the chemokine CXCL12.^15^ We hypothesized the administration of intrauterine CXCL12 would recruit BM stem cells to the uterus and enable uterine repair without BM transplantation. The aim of this study was to determine whether the migration of BMDSCs during endometrial repair is enhanced by CXCL12 in AS, resulting in improved reproductive performance. In this model of AS, mice had smaller litters and took a longer time to conceive. The CXCL12-treated Asherman’s mice had significantly larger litters and a required shorter time to conceive, restoring pregnancy to a level similar to the healthy controls.

Without injury, few bone marrow stem cells are recruited to and engraft in the uterine endometrium. Injury (AS) increased stem cell engraftment to the uterus in the absence of supplemental CXCL12 in both irradiated and non-irradiation mice receiving BM transplant. Treatment with CXCL12 resulted in the highest number of engrafted BMDSCs.

Bone marrow-derived mesenchymal stem cells are undifferentiated, capable of traveling to distant organs and differentiating into non-hematopoietic cells, suggesting that BMDSCs may contribute to tissue repair and regeneration.^17^, ^18^, ^19^, ^20^, ^21^ Recently, new opportunities have arisen in the treatment of reproductive diseases as a result of our understanding of stem cell biology. For the continued survival of most of the other mammalian species, a complete regeneration of endometrium, including the glandular epithelium and stroma, in each reproductive cycle is of vital importance.^17^ Endometrial regeneration can be driven primarily by both endometrial progenitor stem cells; however, it is also supplemented by migration of exogenous bone marrow-derived stem cells.^22^ Our laboratory previously demonstrated endometrial regeneration by stem cells in women diagnosed with leukemia who received bone marrow transplants from a single-HLA antigen mismatched donor.^23^ In endometrial samples of bone marrow recipients, endometrial epithelial and stromal cells of donor origin were detected. We also showed that ischemia/reperfusion injury of uterus promoted BMDSCs migration to the endometrium and characterized the role of BMDSCs in AS.^13^, ^16^ In response to AS, the stem cell recruitment to endometrium was enhanced; as a result, the uterine cavity was re-established and the uterine function was restored.^13^

The endogenous endometrial stem cells that reside in the endometrial basalis layer presumably serve as a source of endometrial regeneration.^24^, ^25^ However AS involves severe damage to the endometrial basal layer, which may result in the destruction of endogenous endometrial progenitor stem cells and the loss of endometrial regeneration ability. In this circumstance, BMDSCs will serve as potential endometrial stem cells, which may constitute a source of cells for endometrial repair. BMDSCs include hematopoietic stem cells (HSCs) and mesenchymal stem cells (MSCs), both of which are actively involved in response to injury.^26^, ^27^ In other tissues, the MSC subpopulation alone takes part in repair following tissue damage.^28^, ^29^ We have demonstrated in a previous study that, in response to injury, BM-derived MSCs are recruited to the endometrium.^16^ Thereby, we have counted CD45^−^ cells to exclude the HSCs and to evaluate MSCs that play a role in the repair of endometrium. This does not eliminate the likely possibility that leukocytes recruited after injury are also part of the repair process. Following their recruitment from bone marrow, MSCs cells serve as a source of progenitor cells as well as secrete trophic factors that aid in tissue repair.^17^ Here, we showed that treatment with CXCL12 resulted in an increase in BMDSCs engraftment of the uterus in AS, leading to improved fertility.

CXCL12 is a ligand of CXCR4 chemokine receptor. Their interaction plays a key role in the mobilization and homing of stem cells.^14^ Expressed on the surface of stem cells, CXCR4 functions to modulate migration.^30^ CXCL12 is mainly produced by stromal cells as well as endothelial cells of various organs including endometrium, skeletal muscle, bone marrow, liver, and brain.^31^ In several other tissues, it has been previously demonstrated that elevated CXCL12 production at an injury site augments the recruitment of stem cells and facilitates functional recovery.^32^, ^33^, ^34^, ^35^ Recently, it is shown that CXCL12 drives migration of stem cells to the endometrium, and this identification may allow therapeutic use in AS to restore fertility.^15^ If a serious injury ensues, as in the case with severe AS, the restricted amount of circulating stem cells may be regarded as a limiting factor in the repair process. The risk of AS is likely exacerbated further by a diminished endogenous endometrial stem cell pool in the damaged endometrium and an altered capability to recruit BM-derived cells to the uterus. Here, we demonstrate increased bone marrow-derived stem cells engraft the murine endometrium with CXCL12 treatment. In concordance with this finding, CXCL12 facilitated remodeling of the uterus, prevented fibrosis and enhanced uterine function (litter size and decreased time to conceive) in the AS model without supplemental BM transplantation. Therefore, in the treatment of severe uterine damage, CXCL12 may be used to mobilize stem cells, eliminating the need for stem cell transplantation.

## Materials and Methods

### Animals

C57BL/6J wild-type and ubiquitin-GFP mice were purchased from Charles River Laboratories and The Jackson Laboratory, respectively. Mice were housed and maintained (four to five per cage) in a room (21 ± 1°C) with a 12-h light/dark cycle (7:00 a.m. to 7:00 p.m.) with ad libitum access to food and water, in the Yale Animal Resources Center (YARC) at Yale School of Medicine. All animal experiments were conducted in accordance with an approval protocol from Institutional Animal Care and Use Committee (IACUC) appointed by Yale University.

### Experimental Groups

To assess the stem cell trafficking to AS, 8-week-old female C57BL/6J wild-type mice (n = 40) were irradiated with two doses of 4.8 Gy, 3 hr apart, monitored for general toxicity and assessed for well-being. The irradiated mice received unfractionated BM cells (1 × 10^7^) from mice expressing GFP as donors by retro-orbital injection. Two weeks after BM transplant for reconstitution of the bone marrow, surgery was performed on these recipient mice to create AS (n = 20) according to a modified version of our validated protocol^13^ or a sham control (n = 20) under inhalation anesthesia (isoflurane; Henry Schein). In the modified protocol, we administered a reduced level of trauma, resulting in less uterine injury than we had previously reported. We induced a milder form of AS that did not cause sterility; rather, it reduced litter size and increased time to conceive but did not prevent pregnancy altogether. Both the AS and sham groups were divided into four sub-groups and each sub-group received one of four treatments: PBS (n = 5), intrauterine CXCL12 (Sigma-Aldrich) (1 μg/mouse, n = 5), subcutaneous AMD3100 (a CXCR4 antagonist; Sigma-Aldrich) (10 mg/kg/day, n = 5), or intrauterine CXCL12 plus subcutaneous AMD3100 (n = 5) injections. In order to ascertain a homogeneous CXCL12 injection to the desired area, the needles that were used to generate endometrial injury were initially embedded into CXCL12 solution before the scratching of the endometrium takes place. For the injection, the syringe needle was sequentially inserted into four walls of the uterus (anterior, posterior, left, and right) along the entire length of the uterus and slowly withdrawn while pushing the piston of the syringe. After three estrus cycles following treatment, animals were euthanized by CO_2_. Uterine horns were collected for analysis of BMDSC engraftment into the uterus. In CXCL12 group, the biopsies were taken only from the injected horns and evaluated. Uterine horns were placed in 4% paraformaldehyde for immunohistochemical analysis for the presence of GFP^+^CD45^−^ and GFP^+^CK^+^ cells.

To eliminate the effect of the irradiation of uterine stem cell recruitment, we also conducted the identical experiments on mice that were not irradiated. These mice were supplemented with unfractionated BM cells (1 × 10^7^) from mice expressing GFP as donors by retro-orbital injection. Although the entire BM is not replaced by GFP^+^ cells, we note cell flux to the uterus at lower levels in this model and could use it to evaluate the relative effects of treatments on BM cell recruitment and engraftment.

To assess the effect of CXCL12, AMD3100, or CXCL12 plus AMD3100 on healing and fibrosis in AS, 40 mice that were not irradiated received 1 × 10^7^ unfractionated bone marrow cells from GFP donors by retro-orbital injection. This allowed us to track the effect of injury on healing in the absence of radiation damage to the uterus. Estrus cycle stage was determined by cytological analysis of vaginal lavage. The mice that were in diestrus were subsequently operated on to create either experimental AS in one-half or a sham surgery was performed. The treatment protocol was administered as described above to the each group (n = 5 per group). Three estrus cycles after treatment, uteri were collected and stem cell engraftment was measured by GFP^+^, CD45^−^, and CK^+^ immunofluorescence as well as histological evidence of fibrosis was evaluated by trichrome and Sirius red stain. Photomicrographs were taken using a Zeiss LSM 710 confocal microscope and a Zeiss Axioplan 2 microscope.

In addition, reproductive studies were carried out to assess the functional effect of CXCL12, AMD3100, or CXCL12 plus AMD3100 on pregnancy after AS. An additional 45 non-irradiated mice were used to determine the pregnancy outcome. The 40 mice were divided into four groups (N = 10) that received the same treatments described above. An additional five healthy mice (no AS) were used as untreated controls. After three estrus cycles, each group was bred with fertile wild-type males. Pregnancy rate, time to conceive, and litter size were calculated after a 90-day breeding period.

### Bone Marrow Cell Isolation and Transplantation

BM was flushed from the humerus, femur, and tibia of 8-week-old GFP male donor mice with cold sterile DMEM: nutrient mixture F-12 (DMEMF-12) (GIBCO, Thermo Fisher Scientific) and filtered through 70-μm cell strainer (BD Biosciences). The yield and viability of BMDSCs were determined by trypan blue staining. Immediately after the uterine damage, unfractionated BM cells in PBS (1 × 10^7^/100 μL) were transplanted into mice by retro-orbital injection.

### Histology and Immunofluorescence

Uterine (horns) tissues were fixed in 4% paraformaldehyde and embedded in paraffin. Five-micrometer tissue sections were mounted on slides. The tissue sections were stained with trichrome and Sirius red for fibrosis according to standard procedures. Masson’s trichrome staining was graded on a scale of 0 to 3 according to the levels of collagen deposition (grade 0, normal; grade 0.5, slight; grade 1, mild; grade 2, moderate; and grade 3, severe).^1^, ^2^ Photomicrographs were taken using a Zeiss Axioplan 2 microscope.

Immunofluorescence studies were carried out to detect the colocalization of GFP-positive bone marrow-derived cells. Tissue sections were deparaffinized by three passages in xylene for 10 min each and subsequent rehydration with graded alcohol, followed by treatment in ammonium chloride (pH 8) against auto-fluorescence for 10 min and washed under tap water for 5 min, and then boiled in sodium citrate buffer at 95°C for 15 min in a steamer for antigen retrieval and blocked by 10% donkey serum for 1 hr for nonspecific antigens. The blocked tissue sections were incubated with the following primary antibodies: polyclonal goat anti-GFP antibody (1:1,000) (ab5450; Abcam), rat anti-CD45 antibody (1:100) (ab25386; Abcam), and rabbit anti-cytokeratin antibody (1:100) (ab9377; Abcam) for overnight at 4°C. The reaction of the secondary antibody was performed with Alexa Fluor 568 donkey anti-goat IgG antibody (A11057), Alexa Fluor 488 donkey anti-rat IgG antibody (A21208), and Alexa Fluor 647 donkey anti-rabbit IgG antibody (A31573) (all are 1:200 dilution and from Life Technologies) for 1 hr at room temperature. Sections were washed in PBS and mounted under coverslips using Vectashield HardSet Mounting Medium with DAPI (Vector Laboratories). Uterine tissue of GFP mice and spleen and skin tissue of wild-type mice were used as positive controls, and each of them demonstrated 100% of the cells staining positive for GFP, CD45, and CK. Immunoreaction with amplification but without primary and/or secondary antibodies was performed as controls. All the visualizations of the slides were done with a laser-scanning confocal microscope (LSM 710; Zeiss) and the ZEN software (Carl Zeiss). Ten high-power fields were counted from each of five sections obtained from each specimen (uterus).

### Statistical Analysis

Data were analyzed using the Statistical Package for the Social Sciences (SPSS), version 17, program (SPSS). Distribution of the variables was investigated using Kolmogorov-Smirnov test. The data with normal distribution were analyzed with one-way ANOVA test, whereas non-normally distributed data were evaluated with Kruskal-Wallis test. When overall significance was observed in ANOVA, pairwise post hoc tests were performed using Tukey’s test, and the Mann-Whitney U test was performed to test the significance of pairwise differences after the Kruskal-Wallis test. Mean values are shown with SEs. Statistical significance was accepted for p < 0.05.

## Author Contributions

G.S.E. designed and conducted all experiments, performed analysis and interpretation of data, and drafted the manuscript. M.M.Z. conducted all experiments and performed analysis and interpretation of data. E.C., I.M., and R.M. conducted all experiments. H.S.T. designed the experiments, performed analysis and interpretation of data, drafted the manuscript, and revised the manuscript for intellectual content.

## Conflicts of Interest

The authors declare no conflict of interest.
